# Heparin and Related Substances for Treating Diabetic Foot Ulcers: A Systematic Review and Meta-Analysis

**DOI:** 10.3389/fendo.2022.749368

**Published:** 2022-02-24

**Authors:** Na Su, Ting Xu, Xiaodan Li, Hanrui Zheng, Bin Wu, Shengzhao Zhang, Yiling Zhou, Liang Du, Yinglan Zhao

**Affiliations:** ^1^ Department of Pharmacology, Key Laboratory of Drug Targeting and Drug Delivery System of the Education Ministry, Sichuan Engineering Laboratory for Plant-Sourced Drug and Sichuan Research Center for Drug Precision Industrial Technology, West China School of Pharmacy, Sichuan University, Chengdu, China; ^2^ Department of Pharmacy, West China Hospital, Sichuan University, Chengdu, China; ^3^ Department of Gastroenterology, West China Hospital, Sichuan University, Chengdu, China; ^4^ Department of Endocrinology and Metabolism, West China Hospital, Sichuan University, Chengdu, China; ^5^ Chinese Cochrane Centre, Chinese Journal of Evidence Based Medicine, West China Hospital, Sichuan University, Chengdu, China

**Keywords:** heparin and related substances, low molecular weight heparin, hyaluronic acid, diabetic foot ulcers, meta-analysis

## Abstract

**Background:**

Diabetic foot ulcers are a major complication of diabetes mellitus (DM), when heparin and heparin related substances may be potentially used as an adjuvant treatment. We aimed to evaluate the efficacy and safety of heparin and heparin related substances for the treatment of diabetic foot ulcers.

**Methods:**

We searched up to March 2021 in the Cochrane Central Register of Controlled Trials (CENTRAL); Ovid MEDLINE; Ovid Embase; EBSCO CINAHL; VIP Chinese Science and Technique Journals Database; China National Knowledge Infrastructure (CNKI) Database and Wan Fang Database investigating heparin or heparin-related substances in patients with diabetic foot ulcers. The primary outcomes included proportion of ulcers completely healed and time to complete ulcer healing. We assessed each included study with the Cochrane ‘Risk of bias’ tool and used the GRADE approach to assess the overall quality of the evidence.

**Results:**

We included nine randomized studies involving 620 participants in the meta-analysis, involving two different heparin and heparin-related substances, low molecular weight heparin (LMWH) and hyaluronic acid. Our study did not show the benefits from LMWH on increasing chance of the ulcer healing (RR: 1.26; 95% CI: 0.78 to 2.04; P=0.35; very low) or shortening the time to complete ulcer healing (SMD: 0.13 d; 95% CI: -0.29 to 0.56; P=0.54; very low). Hyaluronic acid may improve the complete ulcer healing (RR: 1.57; 95% CI: 1.29 to 1.91; P˂0.00001; very low) and shorten the time to complete ulcer healing (SMD -0.84, 95% CI -1.15 to -0.53; P<0.00001; low). Hyaluronic acid and LMWH were generally well tolerated for treating diabetic foot ulcers in this review.

**Conclusion:**

Hyaluronic acid may improve diabetic foot ulcer with very low quality evidence but not LMWH. However, the benefits and harms need further validation in larger trials with different population.

**Systematic Review Registration:**

[https://www.crd.york.ac.uk/prospero/], identifier [PROSPERO, CRD42021269212].

## Introduction

Heparin and related substances are glycosaminoglycans that exist naturally inside the cell and in the extracellular matrix (1, 2). They act by binding selectively to varieties of proteins and pathogens are crucially relevant to many disease processes. These related substances include: low molecular weight heparin (LMWH), chondroitin, heparitin sulphate, hyaluronic acid and keratan sulphate. They have beneficial effects on local tissue microcirculation and oxygenation through the inhibition of thrombin generation and increases in plasma fibrin gel porosity, which may promote vascular perfusion significantly in the peripheral ischemia and healing of chronic ulcers by stimulating production of basic fibroblast growth factor and transforming growth factor-beta 1 (3).

Diabetic foot ulcer is the most serious and costly complication of diabetes mellitus (DM). If the people with diabetic foot ulcer are insensitive to untreated sores and infection, it may lead to ulceration and subsequent limb amputation (4, 5). It occurs in 16% of people with DM and precedes 85% of foot-related amputations, especially in high risk patients (6). There are multiple approaches to treating diabetic foot ulcers that include: glycemic control (7, 8); diet control; conventional therapy for ulcers; correction of arterial insufficiency; resection of the chronic wound (ulcer); and the use of wound dressings (9). However, new clinical treatments are urgently needed for further improve the foot ulcer healing, especially because reconstructive vascular surgery or resection of the chronic wound is not always possible. Recently some published studies proposed that heparin and related substances were benefit for diabetic foot ulcers and confirmed that the positive effects of heparin and related substances on the healing of ulcers (10–12), but most of the studies had methodological limitations. Meanwhile, others suggested that heparin and related substances had no effectiveness (13). Because the effectiveness of treatment is controversial, heparin and related substances was not recommended for treating diabetic foot ulcers by the guidelines. Therefore, we conducted a systematic review of the available evidence for the effects of heparin and heparin related substances for the treatment of diabetic foot ulcer is required.

## Materials and Methods

This systematic review was written based on the Preferred Reporting Items for Systematic Reviews and Meta Analyses (PRISMA). This systematic review was registered on International Prospective Register of Systematic Review (PROSPERO, CRD42021269212).

### Literature Search

We searched the Cochrane Central Register of Controlled Trials (CENTRAL), Ovid MEDLINE, Ovid Embase, EBSCO CINAHL, VIP Chinese Science and Technique Journals Database, China National Knowledge Infrastructure (CNKI) Database, and Wan Fang Database, for articles published up to March 2021, using the keywords: “heparin and related substances”, “diabetic foot ulcers”. Medical Subject Heading (MeSH) was also used during the search when applicable (**Supplementary Information 1**). ClinicalTrials.gov was also searched for unpublished data. The reference lists of included studies and relevant review articles investigating the use of heparin and related substances in diabetic foot ulcers patients are screened for potentially eligible studies.

### Study Selection

Studies were included if they met the following criteria: (1) Participants: participants with type 1 or type 2 DM with foot ulcers of any level of severity. Diagnosis of DM was according to the American Diabetes Association, Report of the Expert Committee on the Diagnosis and Classification of Diabetes Mellitus (14). Otherwise, there was no restriction in relation to the etiology of the ulcer; trials could include ulcers that were neuropathic, ischemic or neuroischemic; (2) Interventions/comparisons: any heparin or heparin-related substance used for treating diabetic foot ulcers, regardless of dosage, route of administration or duration of treatment. Heparin-related substances include: chondroitin, heparitin sulphate, hyaluronic acid and keratan sulphate. We anticipated that likely comparisons would include: heparin or a related substance compared with placebo or conventional therapy. The authors of the included studies defined conventional therapy as standard care for foot ulcers. This included wound care, controlling blood glucose levels, treatment of infection, improving microcirculation, reducing blood pressure, adjusting blood lipids and nutritional support; (3) Outcomes: reporting one of the primary outcomes of interest, namely proportion of ulcers completely healed, time to complete ulcer healing. Our secondary outcomes of interest were amputation and adverse events; (4) Study design: published or unpublished randomized controlled trials (RCTs) and quasi-RCTs.

### Data Extraction

Independently, two review authors (NS, HZ) extracted data from the primary publications and from any associated online appendices using a standardized data extraction form. This extraction form included the following data: the last name of the first author, year of publication, sample size, duration of follow-up, intervention strategy, characteristics of participants (total number; diagnose; location; ulcer area; ulcer duration), funding and outcome.

### Quality Assessment

Independently, two review authors (NS, HZ) assessed each included study with the Cochrane ‘Risk of bias’ tool (15). This included the following items: (1) random sequence generation; (2) allocation concealment; (3) blinding of participants and personnel; (4) blinding of outcome assessment; (5) incomplete outcome data; (6) selective reporting; and (7) other sources of bias. Each of them was judged as low risk, high risk and unclear. Discrepancies were resolved by discussion with a third reviewer (TX). We also used the Grading of Recommendations Assessment, Development and Evaluation (GRADE) method to summarize the evidence profiles, concerning inconsistency, indirectness, imprecision, and other sources of bias.

### Statistical Analyses

We reported estimates for dichotomous data (e.g. ulcer healing rates, adverse events) as risk ratios (RR) with 95% CI. We reported estimates for continuous data (e.g. time to complete ulcer healing) as mean differences (MD) with 95% CI or, if different scales were used, standardized mean differences (SMD) with 95% CI. We considered clinical heterogeneity (that is the degree to which studies vary in terms of participant, intervention and outcome characteristics). Statistical heterogeneity was evaluated using the χ2 test (in which P values less than 0.1 will be considered to indicate significant heterogeneity). Heterogeneity across studies was evaluated using the I^2^ test. Fixed-effect model was used to pool the data from the studies without significant statistical heterogeneity. If the heterogeneity was statistically significant, random-effect model was used and possible sources of heterogeneity were explored. Subgroup analyses according to the duration of follow-up, using that provided in each included study. We defined short-term follow-up as 1 to 12 weeks, long-term follow-up as from > 12 weeks to 24 weeks, and unknown follow-up.

All analyses were carried out using Review Manager 5.3.5 (Copenhagen: The Nordic Cochrane Centre, The Cochrane Collaboration, 2012).

## Results

### Search Results

**Figure 1:**
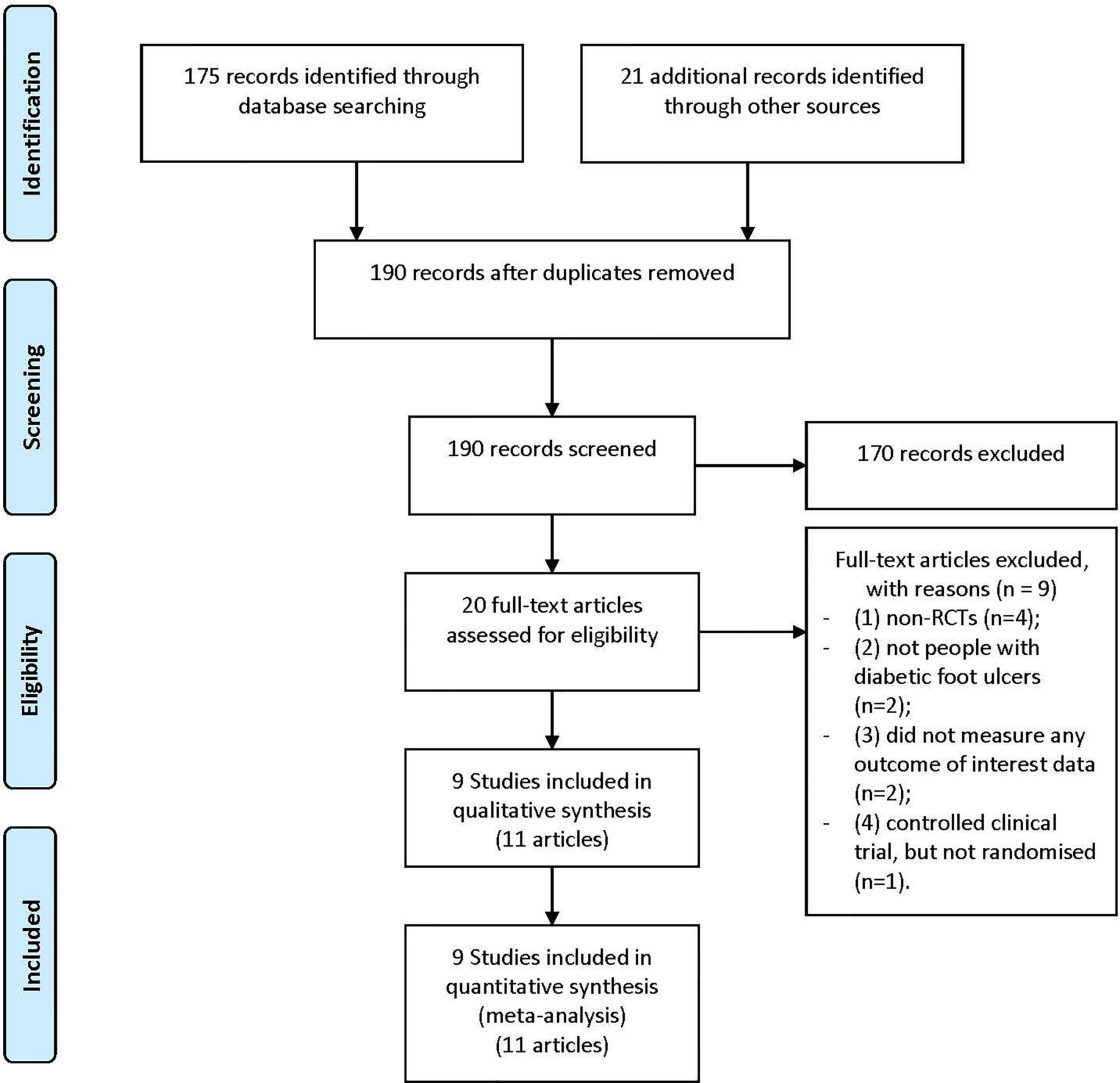
Flow diagram for study identification and inclusion.

Our study selection process is illustrated in **Figure 1**. The primary data search retrieved a total of 196 articles based on the search strategy. After screening the titles and abstracts, 20 potentially eligible papers underwent full-text reviewing. A total of 9 were excluded for the following considerations: (1) non-RCTs (n=4); (2) not people with diabetic foot ulcers (n=2); (3) did not measure any outcome of interest data (n=2); (4) controlled clinical trial, but not randomized (n=1). Eventually, there were 9 studies (11 articles) involving 620 participants (10, 11, 16–22) were included in the final meta-analysis.

### Study Characteristics and Quality Assessment

The characteristics of the studies are shown in **Table 1**. Seven of the included studies were published in English (10, 11, 16, 17, 19, 21, 22), one was published in Chinese (20), and one was published in Korean (18). Three were conducted in Italy (16, 17, 21), one in China (20), one in Sweden (12), three in Korea (18, 19, 22), and one in Spain (13). All included studies were of parallel two-arm design. The minimum size was 25 and the maximum was 160 [19, 21, respectively]. Two of the included studies were all that one study published two articles (10, 11) and seven were all that one study published one article (16–22).

**Table 1:** Baseline characteristics of each included study.

Study	Sample size (I/C)	Location	Patients	Intervention	Control	Follow-up duration (wks)	Ulcer area(I/C) (cm^2^)	Ulcer duration (I/C) (wks)	Funding	Outcomes
Abbruzzese, 2009 (16)	15/15	Italy	Neuropathic foot ulcer with DM	Hyaluronic acid	Conventional therapy	12	2.59 ± 8.8/2.73 ± 10.4	30.8 ± 16.7/22.9± 18.6	None	a, b
Caravaggi, 2003 (17)	43/36	Italy	Foot ulcer with DM	Hyaluronic acid	Conventional therapy	12	5.3 ± 6.76/6.2 ± 7.58	16 ± 40/16 ± 24	Research grant from Fidia Advanced Biopolymers	a,b,d
Eum, 2009 (18)	13/13	Korea	Foot ulcer with DM	Hyaluronic acid	Conventional therapy		3.9 ± 4.38/3.9 ± 2.02	46 ± 94.9/26 ± 20.64	None	a,b
Kalani, 2003 (10)	44/43	Sweden	Diabetic foot ulcers	LMWH	Conventional therapy		NA	NA	Pharmacia Corporation	a,b,c
Lee, 2016 (19)	13/12	Korea	Foot ulcer with DM	Hyaluronic acid	Conventional therapy	24	3.10 ± 2.48/4.80 ± 4.32	18.53 ± 5.82/4.80 ± 4.32	Genewel	a,c,d
Li, 2011 (20)	30/30	China	Foot ulcer with DM	Hyaluronic acid	Conventional therapy		≤6/≤6	2-13/2-13	None	a
Rullan, 2008 (11)	37/33	Spain	Foot ulcer with DM	LMWH	Conventional therapy	12	1.63/1.57	NA	The Primary Health Care Management of Mallorca	a, d
Uccioli, 2011 (21)	80/80	Italy	Foot ulcer with DM	Hyaluronic acid	Conventional therapy	20	8.8 ± 9.4/6.7 ± 7.7	7.4 ± 6.6/7.3 ± 7.8	Anika Therapeutics srl	a,b,d
You, 2014 (22)	31/32	Korea	Foot ulcer with DM	Hyaluronic acid	Conventional therapy	12	3.5 ± 3.7/2.9 ± 2.7	24.4 ± 65.6/24.8 ± 78.8	None	a,b,d

DM, diabetes mellitus; LMWH, low molecular weight heparin; I, intervention; C, control; a, proportion of ulcers completely healed; b, time to complete ulcer healing; c, amputation; d, adverse events; NA, not applicable.

Only one study did not report the age of participants (17), while the other trial reports indicated that age of participants ranged from 38 to 83 years of age. Five studies reported the gender of participants at randomization (10, 11, 18, 19, 22); The proportion of men was reported to be 70.63% (190 to 269). Seven studies described the ulcer area (11, 16–19, 21, 22); the minimum area was 1.57 cm^2^ and the maximum was 8.8 cm^2^ [11, 21, respectively]. Seven studies reported the ulcer duration (16–22), which ranged from two to 30.8 weeks.

Low-molecular-weight heparin (LMWH) was used in two studies (10, 11), and hyaluronic acid was used in seven studies (16–22). No other heparin or heparin-related substances were used in the treatment of diabetic foot ulcers in these trials.

Seven studies reported an adequate sequence generation and we judged them to have a low risk of bias for this domain (10, 11, 16, 17, 19, 21, 22), mostly based on the fact that all of them used computer-generated randomization sequences. The remaining two RCTs did not describe the method of sequence generation used and we judged them to have an unclear risk of bias (18, 20). Five studies reported adequate allocation concealment and we judged them to have a low risk of bias for this domain (10, 11, 16, 19, 21). Four studies described using sealed packages, boxes or envelopes (10, 11, 19, 21), while one study reported that it was difficult to distinguish between the appearance of two groups’ interventions (16). Three studies reported using a method to blind participants and personnel and we judged them to have a low risk of bias (10, 11, 16). Five studies reported using a method to blind outcome assessment and we judged them to have a low risk of bias too (10, 11, 16, 19, 21). The quality assessment results were shown in **Supplementary Figures 1** and **2**.

### LMWH Versus Conventional Therapy

#### Meta-Analyses of Proportion of Ulcers Completely Healed

**Figure 2:**
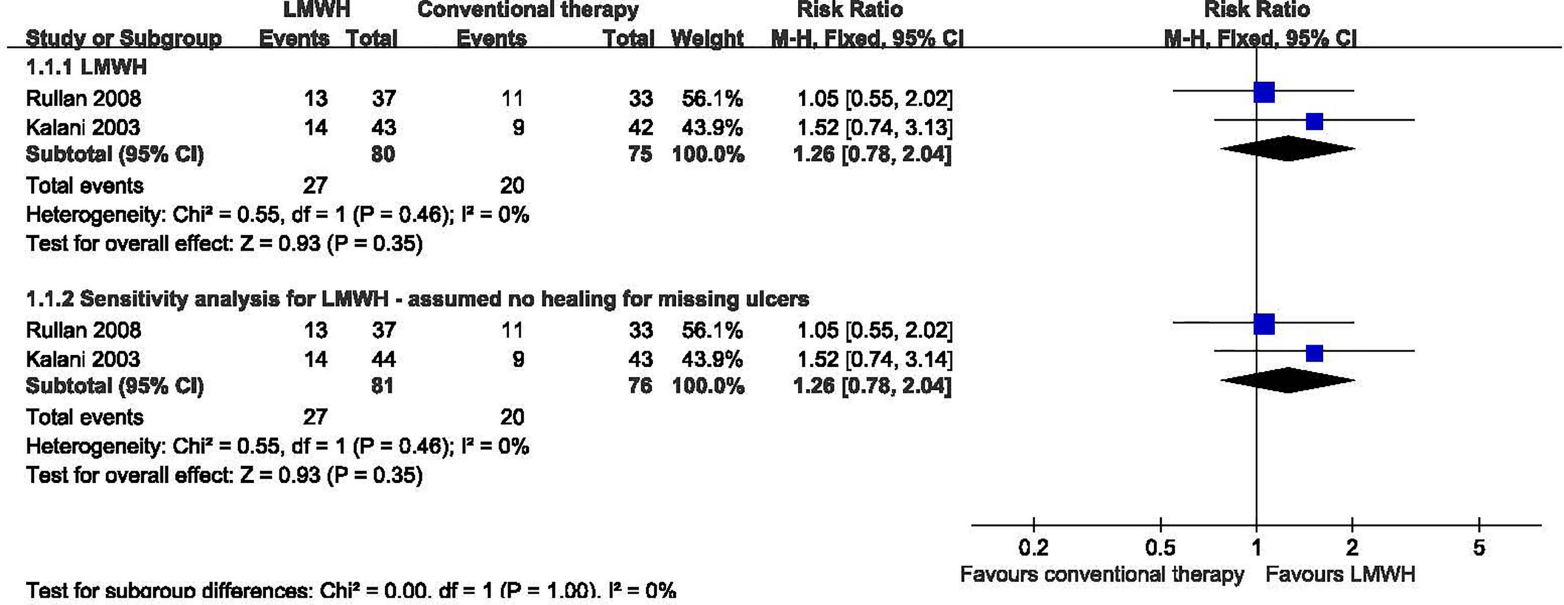
Proportion of ulcers completely healed in patients receiving LMWH versus conventional therapy. LMWH, Low molecular weight heparin; CI, confidence interval; M-H, Mantel-Haenszel.

Two studies with a total of 155 participants (80 participants in LMWH group and 75 in the conventional therapy group) reported the proportion of ulcers completely healed (10, 11). We used the fixed-effect model in the analysis. LMWH was not associated with a statistically significant increase in the proportion of ulcers completely healed (RR: 1.26; 95% CI: 0.78 to 2.04; P=0.35) (**Figure 2**). This was very low quality evidence downgraded one level for risk of bias, one level for imprecision and one level for publication bias (**Table 2**).

**Table 2:** The GRADE profiles: LMWH compared to conventional therapy for diabetic foot ulcer.

Outcomes	Illustrative comparative risks* (95% CI)		Relative effect(95% CI)	No of Participants(studies)	Quality of the evidence(GRADE)	Comments
	Assumed risk	Corresponding risk				
	Control	LMWH versus conventional therapy				
Proportion of ulcers completely healed during follow up - LMWH	267 per 1000	336 per 1000 (208 to 544)	RR 1.26 (0.78 to 2.04)	155 (2 studies)	very low^1,2,3^	
Time to complete ulcer healing (day)		The mean time to complete ulcer healing (day) in the intervention groups was 0.13 standard deviations higher (0.29 lower to 0.56 higher)		85 (1 study)	very low^2,4,5^	SMD 0.13 (-0.29 to 0.56)
Amputation at the end of follow-up	120 per 1000	38 per 1000 (11 to 136)	RR 0.32 (0.09 to 1.13)	155 (2 studies)	very low^1,2,3^	
Total adverse events	303 per 1000	379 per 1000 (194 to 733)	RR 1.25 (0.64 to 2.42)	70 (1 study)	low^2,6^	
Serious adverse events	212 per 1000	161 per 1000 (62 to 435)	RR 0.76 (0.29 to 2.05)	70 (1 study)	low^2,6^	
Bleeding	30 per 1000	27 per 1000 (2 to 415)	RR 0.89 (0.06 to 13.7)	70 (1 study)	low^2,6^	

*The basis for the assumed risk (e.g. the median control group risk across studies) is provided in footnotes. The corresponding risk (and its 95% confidence interval) is based on the assumed risk in the comparison group and the relative effect of the intervention (and its 95% CI).

CI, Confidence interval; RR, Risk ratio; LMWH, Low molecular weight heparin; GRADE, Grading of Recommendations Assessment, Development and Evaluation; Low quality, further research is very likely to have an important impact on our confidence in the estimate of effect and is likely to change the estimate; Very low quality, we are very uncertain about the estimate.

^1^Downgraded one level for risk of bias (High risk of bias for allocation concealment and blinding).

^2^Downgraded one level for imprecision (Very small samples sizes in each study).

^3^Downgraded one level for publication bias [Studies were funded by pharmaceutical companies, such as Pharmacia Corporation (Kalani 2003), Plasticos Rovi S.A.(Rullan 2008)].

^4^Downgraded one level for risk of bias (No ITT analysis was reported).

^5^Downgraded one level for publication bias (Study was funded by Pharmacia Corporation).

^6^Downgraded one level for publication bias (Study was funded by Plasticos Rovi S.A).

In a sensitivity analysis for one study (10), two participants dropped out during the study, one in the LMWH group, the other one in the conventional therapy group. We assumed the missing patients did not heal, but this analysis showed no significant difference. We found no significant heterogeneity among RCTs (I^2^ = 0%, P = 0.46). We used the fixed-effect model in the analysis: RR 1.26, 95% CI 0.78 to 2.04; P=0.35.

Subgroup analyses were carried out according to different duration of follow-up (**Supplementary Table 2**). In the two subgroups (short term follow-up; unknown follow-up period), LMWH was not associated with a statistically significant increase in the proportion of ulcers completely healed after short term follow-up and unknown follow up period.

#### Meta-Analyses of Time to Complete Ulcer Healing

**Figure 3:**
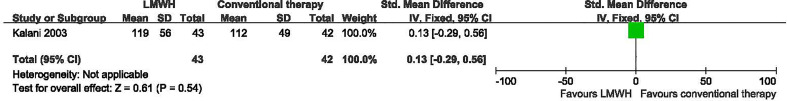
Time to complete ulcer healing in patients receiving LMWH versus conventional therapy. LMWH, Low molecular weight heparin; CI, confidence interval; IV, inverse variance; SD, standardized deviation.

One study with a total of 85 participants (43 participants in LMWH and 42 in the conventional therapy group) reported data on time to complete ulcer healing (10). LMWH was not associated with a statistically significant reduction in the time to complete ulcer healing (SMD: 0.13 d; 95% CI: -0.29 to 0.56; P=0.54) (**Figure 3**). This was very low quality evidence downgraded three times for risk of bias, imprecision and publication bias (**Table 2**).

#### Meta-Analyses of Amputation

**Figure 4:**
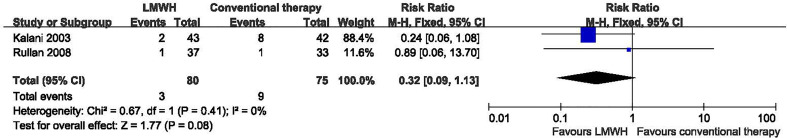
Amputation in patients receiving LMWH versus conventional therapy. LMWH, Low molecular weight heparin; CI, confidence interval; M-H, Mantel-Haenszel.

Two studies with a total of 155 participants (80 participants in LMWH group and 75 in the conventional therapy group) reported on amputation (10, 11). We found no significant heterogeneity between the RCTs (I^2^ = 0%, P = 0.41), so used the fixed-effect model in the analysis. LMWH was not associated with a statistically significant reduction in the proportion of amputations (RR: 0.32; 95% CI: 0.09 to 1.13; P=0.08) (**Figure 4**). This was very low quality evidence downgraded three times for risk of bias, imprecision and publication bias (**Table 2**).

Subgroup analyses were carried out according to different duration of follow-up (**Supplementary Table 2**). In the two subgroups (short term follow-up; unknown follow-up period), LMWH was not associated with a statistically significant reduction in the proportion of amputations after short term follow-up and unknown follow up period.

#### Meta-Analyses of Adverse Events

Only one study with a total of 70 participants (37 participants in LMWH group and 33 in the conventional therapy group) reported adverse events (11). The adverse events occurred in the LMWH group and included bleeding, amputation and death. Analysis of this trial provided the following results: for total adverse events (RR: 1.25; 95% CI: 0.64 to 2.42; P=0.51); for serious adverse events (RR: 0.76; 95% CI: 0.29 to2.05; P=0.59); and that the bleeding situation was not significantly better with LMWH treatment than that with conventional therapy (RR: 0.89; 95% CI: 0.06 to 13.70; P=0.93). It was clear that there was no increase in total adverse events or serious adverse events in patients with LMWH compared with conventional therapy.

### Hyaluronic Acid Versus Conventional Therapy for Treating Diabetic Foot Ulcers

#### Meta-Analyses of Proportion of Ulcers Completely Healed

**Figure 5:**
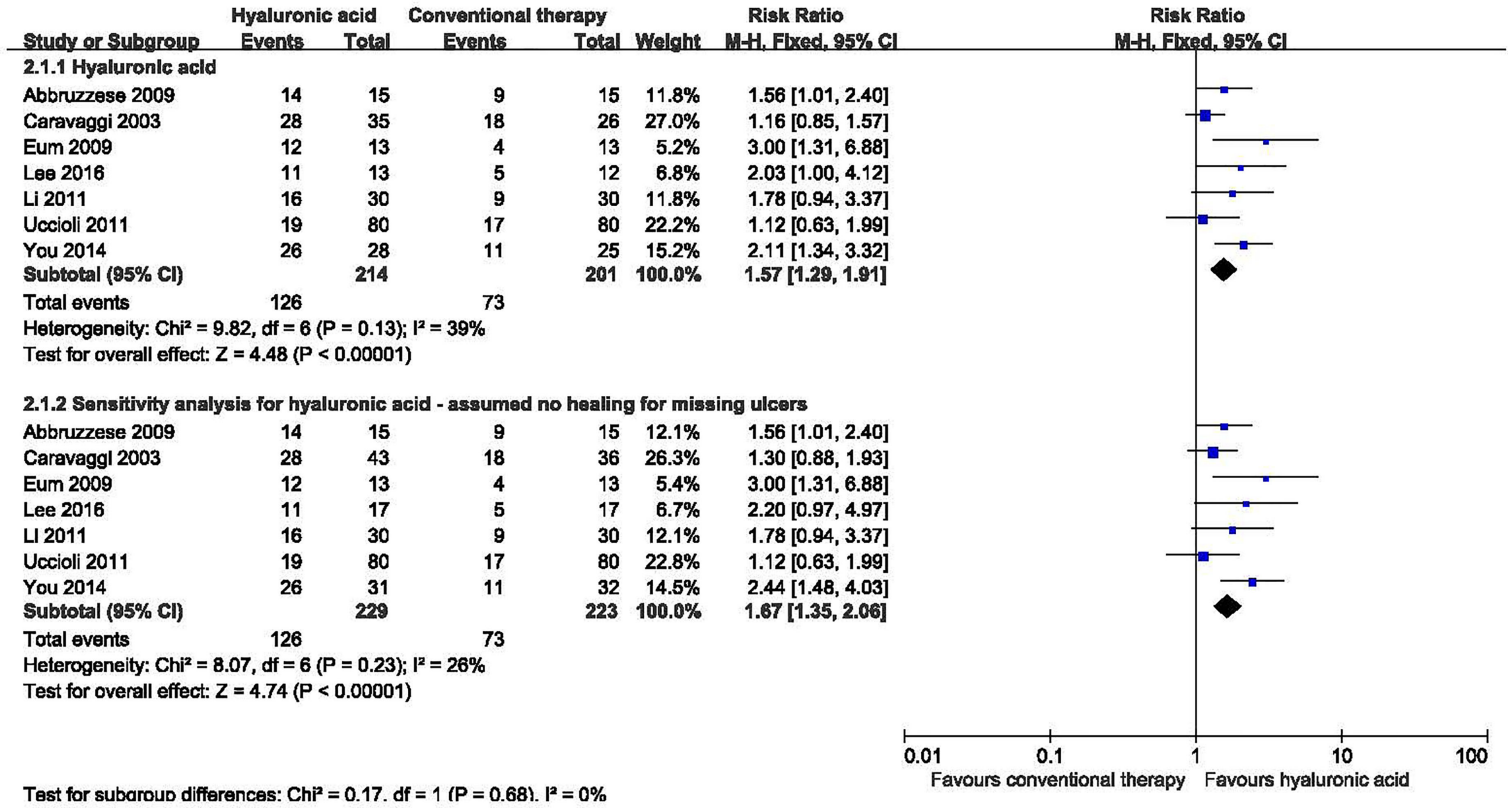
Proportion of ulcers completely healed in patients receiving hyaluronic acid versus conventional therapy. CI, confidence interval; M-H, Mantel-Haenszel.

Seven studies with a total of 415 participants (214 participants in hyaluronic acid group and 201 in the conventional therapy group) reported the proportion of ulcers completely healed (16–22). Hyaluronic acid was associated with a statistically significant increase in the proportion of ulcers completely healed. There was moderate heterogeneity among these studies (I^2^ = 39%, P = 0.13), and a fixed-effect model was used in the analysis (RR: 1.57; 95% CI: 1.29 to 1.91; P˂0.00001) (**Figure 5**). This was very low quality evidence downgraded three times for risk of bias, imprecision and publication bias (**Table 3**).

**Table 3:** The GRADE profiles: hyaluronic acid compared to conventional therapy for diabetic foot ulcer.

Outcomes	Illustrative comparative risks* (95% CI)		Relative effect(95% CI)	No of Participants(studies)	Quality of the evidence(GRADE)	Comments
	Assumed risk	Corresponding risk				
	Control	Hyaluronic acid versus conventional therapy				
Proportion of ulcers completely healed during follow up - Hyaluronic acid	361 per 1000	567 per 1000 (466 to 690)	RR 1.57 (1.29 to 1.91)	415 (7 studies)	very low^1,2^	
Time to complete ulcer healing (day)		The mean time to complete ulcer healing (day) in the intervention groups was 0.84 standard deviations lower (1.15 to 0.53 lower)		179 (4 studies)	low^1,3^	SMD -0.85 (-1.15 to -0.54)
Amputation at the end of follow-up	20 per 1000	94 per 1000 (0 to 1000)	RR 4.67 (0.02 to 893.31)	196 (2 studies)	very low^1,2,4,5^	
Total adverse events	176 per 1000 129 per 1000	129 per 1000 (30 to 556)	RR 0.73 (0.17 to 3.15)	234 (2 studies)	very low^1,3,6^	
Serious adverse events - Hyaluronic acid	123 per 1000	101 per 1000 (25 to 411)	RR 0.82 (0.2 to 3.34)	265 (3 studies)	very low^1,2,3,7^	

*The basis for the assumed risk (e.g. the median control group risk across studies) is provided in footnotes. The corresponding risk (and its 95% confidence interval) is based on the assumed risk in the comparison group and the relative effect of the intervention (and its 95% CI).

CI, Confidence interval; RR, Risk ratio; GRADE, Grading of Recommendations Assessment, Development and Evaluation; Low quality, further research is very likely to have an important impact on our confidence in the estimate of effect and is likely to change the estimate; Very low quality, we are very uncertain about the estimate.

^1^Downgraded one level for imprecision (Very small samples sizes in each study).

^2^Downgraded one level for publication bias [Study was funded by Genewel (Seoul, South Korea)].

^3^Downgraded one level for risk of bias (High risk of bias for allocation concealment and blinding).

^4^Downgraded one level for risk of bias (High risk of bias for blinding of participants and personnel, no ITT analysis was reported).

^5^Downgraded one level for inconsistency (Substantial heterogeneity was present among the studies(I2 = 88%, P=0.003). One study’conclusion was contrary to another).

^6^Downgraded one level for inconsistency (Substantial heterogeneity was present among the studies(I2 = 70%, P=0.07). One study’conclusion was contrary to another).

^7^Downgraded one level for inconsistency (Substantial heterogeneity was present among the studies(I2 = 65%, P=0.06). One study’conclusion was contrary to another).

We performed a sensitivity analysis for three studies in which there had been substantial dropouts (17, 19, 22). One study reported that 10 participants from the control group and eight from the treatment group withdrew before completion of the study (17); Ten participants did not complete the treatment in one study (17); And nine participants were lost to follow up in one study (19). We assumed the missing participants did not heal, this analysis showed no significant difference. No significant heterogeneity was found in the sensitivity analysis (I^2^ = 26%, P = 0.23), and a fixed-effect model was used in the analysis. Hyaluronic acid was associated with a statistically significant increase in the proportion of ulcers completely healed in the sensitivity analysis (RR: 1.67; 95% CI: 1.35 to 2.06; P<0.00001).

Subgroup analyses were carried out according to different duration of follow-up (**Supplementary Table 2**). In the three subgroups (short term follow-up; long term follow-up; unknown follow-up period), Hyaluronic acid was associated with a statistically significant increase in the proportion of ulcers completely healed after short term follow-up (RR: 1.51; 95% CI: 1.05 to 2.17; P=0.03) and unknown follow-up period (RR: 2.12; 95% CI: 1.40 to 3.20; P=0.0004).

#### Meta-Analyses of Time to Complete Ulcer Healing

**Figure 6:**
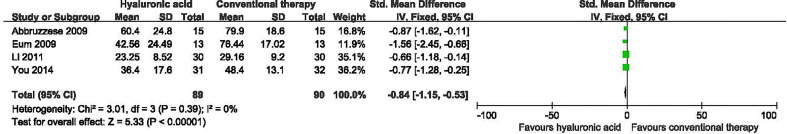
Time to complete ulcer healing in patients receiving hyaluronic acid versus conventional therapy. CI, confidence interval; IV, inverse variance; SD, standardized deviation.

Four studies with a total of 179 participants (89 participants in hyaluronic acid group and 90 in the conventional therapy group) reported the data on time to complete ulcer healing (16, 18, 20, 22). No significant heterogeneity was found among these studies (I^2^ = 0%, P = 0.39), and a fixed-effect model was used in the analysis. Hyaluronic acid was associated with a statistically significant reduction in the time to complete ulcer healing (SMD -0.84, 95% CI -1.15 to -0.53; P<0.00001) (**Figure 6**). This was low quality evidence downgraded two times for risk of bias and imprecision (**Table 3**).

Subgroup analyses were carried out according to different duration of follow-up (**Supplementary Table 2**). In the two subgroups (short term follow-up; unknown follow-up period), Hyaluronic acid was associated with a statistically significant reduction in the time to complete ulcer healing after short term follow-up (SMD -0.84, 95% CI -1.22 to -0.37; P=0.0002) and unknown follow-up period (SMD -1.03, 95% CI -1.90 to -0.16; P=0.02).

#### Meta-Analyses of Amputation

**Figure 7:**
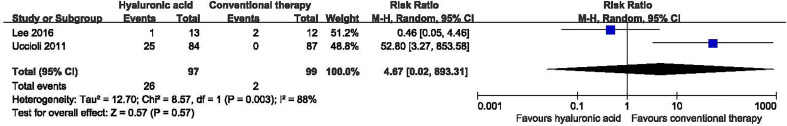
Amputation in patients receiving hyaluronic acid versus conventional therapy. CI, confidence interval; M-H, Mantel-Haenszel.

Two studies with a total of 196 participants (97 participants in hyaluronic acid group and 99 in the conventional therapy group) reported on amputation at the end of follow-up (19, 21). Hyaluronic acid was not associated with a statistically significant reduction in the proportion of amputations (RR 1.26, 95% CI 0.12 to 13.46; P=0.85) (**Figure 7**).

Subgroup analyses were carried out according to different duration of follow-up (**Supplementary Table 2**). Hyaluronic acid was not associated with a statistically significant reduction in the proportion of amputations after long term follow-up and unknown follow up period.

#### Meta-Analyses of Adverse Events

Five studies reported adverse events (16, 17, 19, 21, 22). The adverse events that occurred in the hyaluronic acid group were infections (16), pain (16), skin sensitization (16), wound infections (22). The adverse events that occurred in the conventional therapy group were infections (16), pain (16), skin sensitization (16), odor (16), wound infections (22), lower limb fracture (22), upper respiratory tract infection (22). Two studies with a total of 234 participants (115 participants in the hyaluronic acid group and 119 in the conventional therapy group) reported total adverse events (21, 22). Substantial heterogeneity was present among the studies (I^2^ = 70%, P = 0.07), and so we used the random-effects model for analysis. Hyaluronic acid was not associated with a statistically significant increase in adverse events (RR 0.73, 95% CI 0.17 to 3.15; P=0.67). Three studies with a total of 265 participants (135 participants in the hyaluronic acid group and 130 in the conventional therapy group) reported serious adverse events (17, 19, 21). Substantial statistical heterogeneity was present among the studies (I^2^ = 65%, P = 0.06), and so we used a random-effects model in the analysis. Hyaluronic acid was not associated with a statistically significant increase in serious adverse events (RR 0.82, 95% CI 0.20 to 3.34; P=0.78). We carried out a sensitivity analysis for three studies (17, 19, 21). We assumed the missing participants were free of serious adverse events, this analysis showed no significant difference between groups (RR 0.85, 95% CI 0.22 to 3.38; P=0.82).

Subgroup analyses in adverse events and serious adverse events were carried out according to different duration of follow-up (**Supplementary Table 2**). In the two subgroups (short term follow-up; long term follow-up), hyaluronic acid was not associated with a statistically significant increase in adverse events. In the three subgroups (short term follow-up; long term follow-up; unknown follow-up period), hyaluronic acid was not associated with a statistically significant increase in serious adverse events.

## Discussion

The interventions investigated were LMWH and hyaluronic acid in this review. We did not find trials that evaluated other heparin or heparin-related substances as interventions in the treatment of diabetic foot ulcers. This systematic review involving 620 individuals showed that hyaluronic acid may increase the proportion of ulcers completely healed and decreasing the time to complete healing. The result is similar to that of a previous meta-analysis, which showed hyaluronic acid is beneficial in increasing the rate of diabetic foot healing (P = 0.047) compared with controls (23). But we found no evidence that showed that LMWH could increase the ulcer healing or shortening the time to ulcer healing. Similarly, there was no evidence of a beneficial effect of LMWH on any of the other outcomes, such as amputation. The subgroup analyses suggested that the ulcers healing effect did not change with the duration of follow-up, whether hyaluronic acid or LMWH. Hyaluronic acid and LMWH were generally well tolerated for treating diabetic foot ulcers in this review. The adverse events in the heparin or heparin-related substances groups were similar to controls. There was no increasing significantly in bleeding in LMWH.

LMWHs are a class of anticoagulants that includes enoxaparin, nadroparin, dalteparin, tinzaparin, bemiparin, and reviparin (24). Studies have claimed that heparin can improve hemorheological parameters, increase arterial blood supply and enhance healing in patients with diabetic foot ulcers (25–27). Firstly, LMWH has more favorable bioavailability and pharmacokinetics which means it can be administered subcutaneously without monitoring. Secondly, LMWH may result in fewer bleeding complications due to a less pronounced effect on platelet function and vascular permeability (28), which means that it can be used long-term as an out-of-hospital treatment because of its relative safety. Subcutaneous injection of dalteparin, one type of LMWH, can improve the capillary circulation in the ulcer margin, which positively influences the healing process of chronic foot ulcers in diabetic patients (29). Hyaluronic acid is a GAG distributed widely throughout connective, epilethial and neural tissues. Recent studies showed nasal filling with hyaluronic acid could treat septal ulcer (30), and hyaluronic acid sodium salt 0.2% gel could treat recalcitrant distal leg ulcer (31). In this review we have observed hyaluronic acid may improve diabetic foot ulcer but not LMWH, The reason may be that the sample sizes and the number of included studies on LMWH were too small to permit adequate assessment of this intervention, and no studies referred to a sample size calculation. In addition, subgroup analyses did not do according to the ulcer stage. Rullan proved there were differences for bemiparin between subgroups in terms of age, the number of smokers, and the distribution of grade I and grade II ulcers (11). Though the experimental results proposed that LMWH may be benefit for diabetic foot ulcers, the baselines of patients were different in practice and there was a lot of uncertainty.

Although there has been systematic review published before (23), this is the first study focusing on the efficacy and safety of heparin and heparin related substances for diabetic foot ulcers. The heterogeneity between each included study was no significant, however clinical heterogeneity may also exist, including the use of varying types and duration of follow-up of heparin and heparin related substances among the studies or other baseline and clinical characteristics of the individuals recruited. For example, the majority of the study population was men.

Our research had several limitations. Firstly, we intended to include people with type 1 or type 2 diabetic foot ulcers according to the American diabetes association (ADA). However, only one study described participants with DM according to the diagnostic criteria of the ADA 1997; the remaining included studies did not describe the diagnostic criteria they used for DM. Secondly, although the studies used the Wagner grading system for ulcers (32), the stage of ulcer recruited to different studies varied. In seven studies the ulcer stage of included participants was Wagner grade 1-2 (10, 11, 17–19, 21, 22); in one studies the ulcer stage was Wagner grade 1-3 (20); and in another the authors did not define the eligible Wagner grade (16). Therefore, there may be potential bias in the selection of participants. Thirdly, most studies were funded by pharmaceutical companies, such as Pharmacia Corporation (10), Plasticos Rovi S.A (11), which means that a potential commercial bias cannot be excluded from this review. And finally, the results were still less significant different, especially LMWH. Therefore, further studies with larger populations and longer follow-up periods are needed.

In conclusion, Current evidence suggests that hyaluronic acid could be a potentially beneficial therapy that promotes the healing of diabetic foot ulcers compared with conventional therapy, and that better methods of administration would be to use dressings of an autologous fibroblast-hyaluronic acid complex and hyaluronic acid gel. However, the low, or very low, quality of the current evidence means that this conclusion may be substantially affected by any future studies. Meta-analysis of trials that compared treatment with LMWH to conventional ulcer therapy showed no significant improvement for either treatment regimen. Therefore, it is recommended that hyaluronic acid may be used for diabetic foot ulcer but not LMWH. In addition, high quality randomized controlled trials are needed to assess the effects of LMWH for diabetic foot ulcers.

## Data Availability Statement

The datasets presented in this study can be found in online repositories. The names of the repository/repositories and accession number(s) can be found in the article/**Supplementary Material**.

## Author Contributions

Design: NS and YZhao. Data curation: NS, HZ, and TX. Formal analysis: NS and XL. Methodology: BW and LD. Writing manuscript: NS, TX, XL, HZ, BW, SZ, YZhou, LD, and YZhao. All authors contributed to the article and approved the submitted version.

## Funding

NS was supported by grants from Health Commission Program (grant number 2020-111) and 1.3.5 Project for Disciplines of Excellence, West China Hospital, Sichuan University (grant number 2018HXFH048).

## Conflict of Interest

The authors declare that the research was conducted in the absence of any commercial or financial relationships that could be construed as a potential conflict of interest.

## Publisher’s Note

All claims expressed in this article are solely those of the authors and do not necessarily represent those of their affiliated organizations, or those of the publisher, the editors and the reviewers. Any product that may be evaluated in this article, or claim that may be made by its manufacturer, is not guaranteed or endorsed by the publisher.

## Supplementary Material

Supplementary Figure 1 Risk of bias graph of all included studies (n = 9).

Supplementary Figure 2 Risk of bias summary of all included studies (n = 9).
