# Supplementary figures and images for: Heparin and Related Substances for Treating Diabetic Foot Ulcers: A Systematic Review and Meta-Analysis
Source: Front Endocrinol (Lausanne). 2022 Feb 24;13:749368. doi: 10.3389/fendo.2022.749368 (PMC8907383; doi:10.3389/fendo.2022.749368)

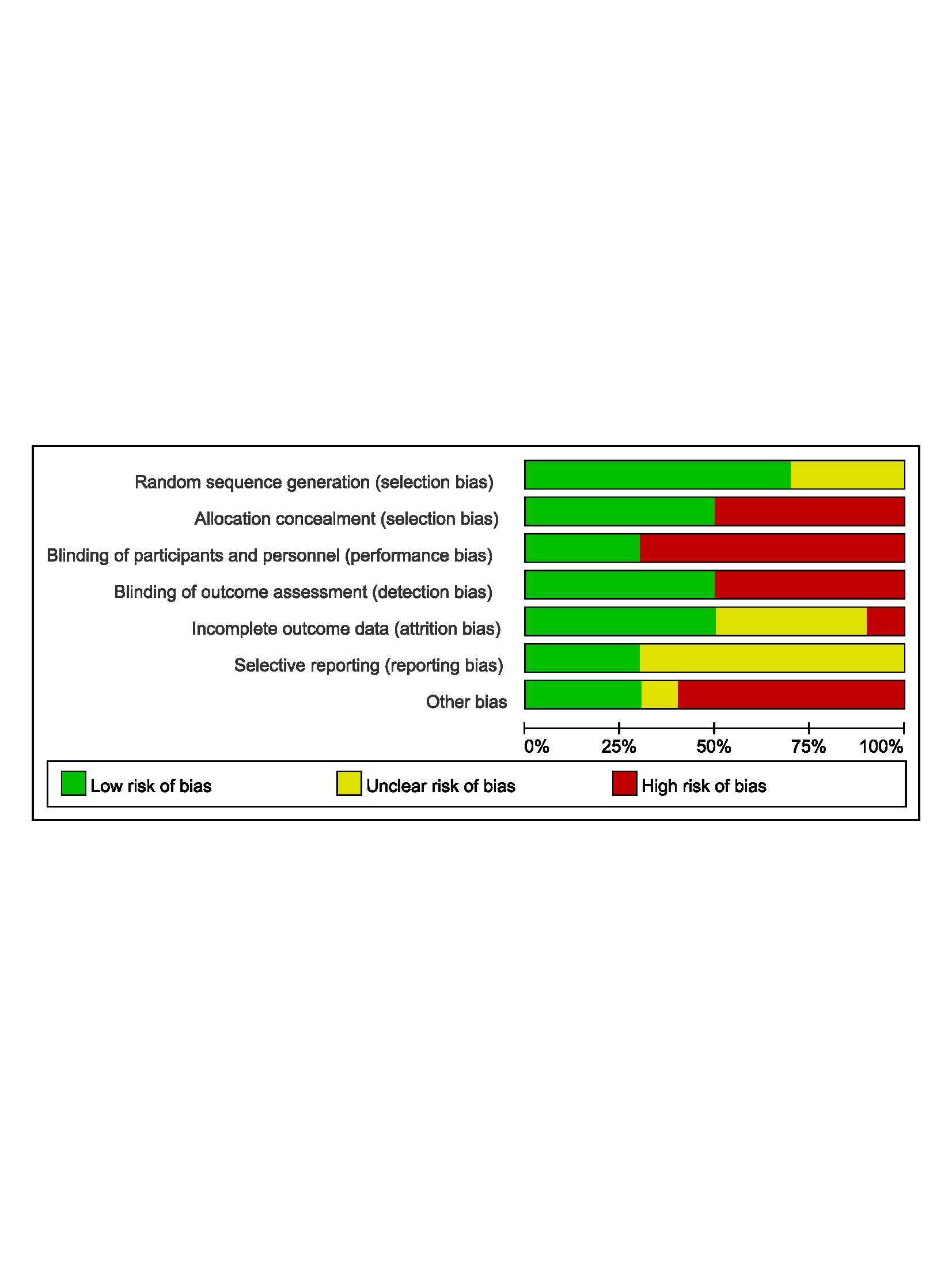

Supplement: Supplementary Figure 1 — Risk of bias graph of all included studies (n = 9). [file Image_1.jpeg]

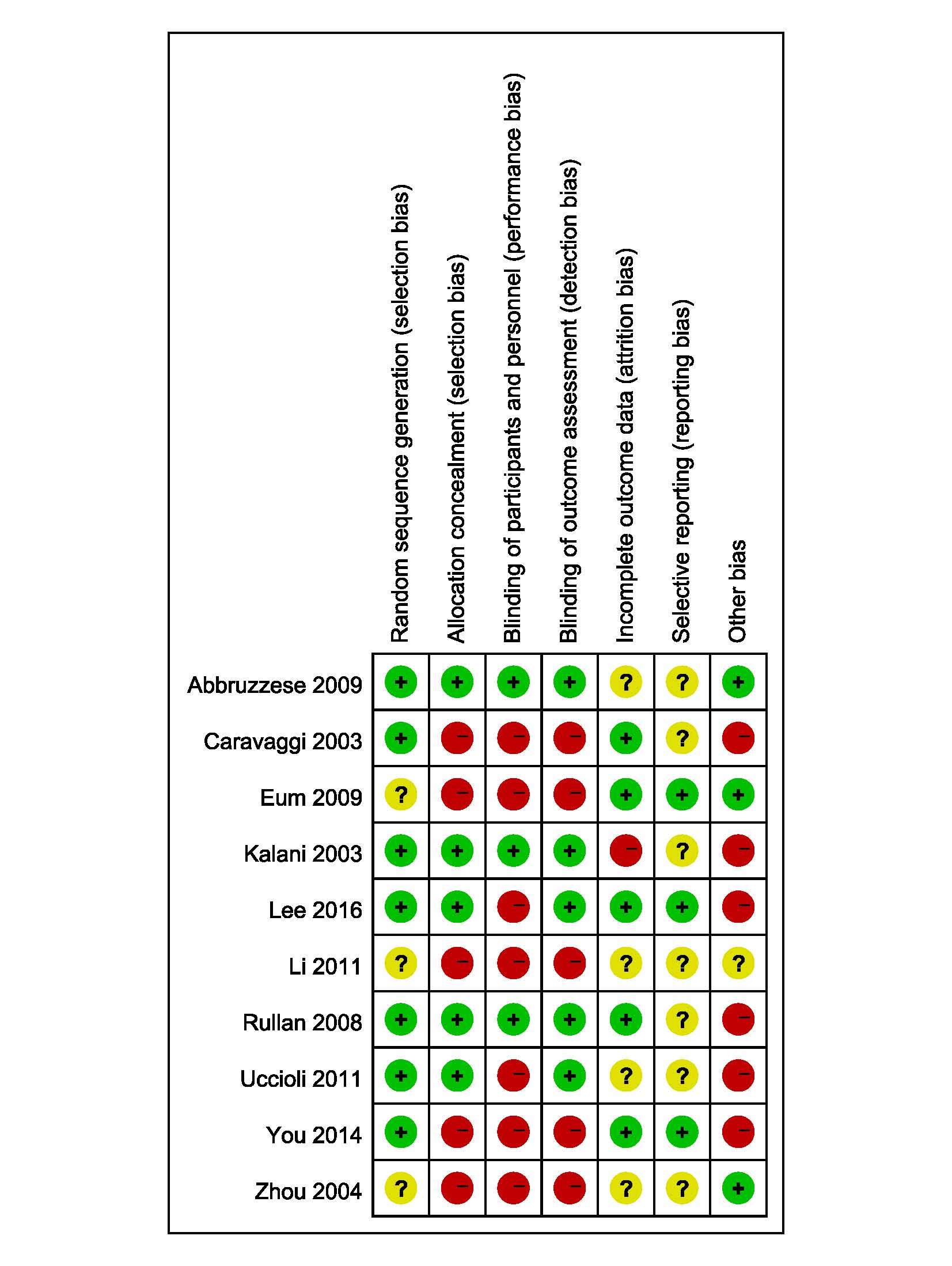

Supplement: Supplementary Figure 2 — Risk of bias summary of all included studies (n = 9). [file Image_2.jpeg]
