# Supplementary material for: Heparin and Related Substances for Treating Diabetic Foot Ulcers: A Systematic Review and Meta-Analysis
Source: Front Endocrinol (Lausanne). 2022 Feb 24;13:749368. doi: 10.3389/fendo.2022.749368 (PMC8907383; doi:10.3389/fendo.2022.749368)
Supplement: Supplementary file 4 [file Table_2.docx]

**Supplementary Table 2 Subgroup analysis of primary outcome**

**LMWH versus conventional therapy**

| **Outcome or Subgroup** | **Studies** | **Participants** | **Statistical Method** | **Effect Estimate** |
| --- | --- | --- | --- | --- |
| **1.1 Proportion of ulcers completely healed** | 2 | 155 | Risk Ratio (M-H, Fixed, 95% CI) | 1.26 [0.78, 2.04] |
| **1.1.1 Short term follow-up** | 1 | 70 | Risk Ratio (M-H, Fixed, 95% CI) | 1.05 [0.55, 2.02] |
| **1.1.2 Unknown follow-up period** | 1 | 85 | Risk Ratio (M-H, Fixed, 95% CI) | 1.52 [0.74, 3.13] |
| **1.2 Proportion of ulcers completely healed (Sensitivity analysis)** | 2 | 157 | Risk Ratio (M-H, Fixed, 95% CI) | 1.26 [0.78, 2.04] |
| **1.3 Time to complete ulcer healing (day)** | 1 | 85 | Std. Mean Difference (IV, Fixed, 95% CI) | 0.13 [-0.29, 0.56] |
| **1.4 Amputation** | 2 | 155 | Risk Ratio (M-H, Fixed, 95% CI) | 0.32 [0.09, 1.13] |
| **1.4.1 Short term follow-up** | 1 | 70 | Risk Ratio (M-H, Fixed, 95% CI) | 0.89 [0.06, 13.70] |
| **1.4.2 Unknown follow-up period** | 1 | 85 | Risk Ratio (M-H, Fixed, 95% CI) | 0.24 [0.06, 1.08] |
| **1.5 Total adverse events** | 1 | 70 | Risk Ratio (M-H, Fixed, 95% CI) | 1.25 [0.64, 2.42] |
| **1.6 Serious adverse events** | 1 | 70 | Risk Ratio (M-H, Fixed, 95% CI) | 0.76 [0.29, 2.05] |
| **1.7 Bleeding** | 1 | 70 | Risk Ratio (M-H, Fixed, 95% CI) | 0.89 [0.06, 13.70] |

## Hyaluronic acid versus conventional therapy

| **Outcome or Subgroup** | **Studies** | **Participants** | **Statistical Method** | **Effect Estimate** |
| --- | --- | --- | --- | --- |
| **2.1 proportion of ulcers completely healed** | 7 | 415 | Risk Ratio (M-H, Random, 95% CI) | 1.60 [1.24, 2.05] |
| **2.1.1 Short term follow-up** | 3 | 144 | Risk Ratio (M-H, Random, 95% CI) | 1.51 [1.05, 2.17] |
| **2.1.2 Long term follow-up** | 1 | 160 | Risk Ratio (M-H, Random, 95% CI) | 1.12 [0.63, 1.99] |
| **2.1.3 Unknown follow-up period** | 3 | 111 | Risk Ratio (M-H, Random, 95% CI) | 2.12 [1.40, 3.20] |
| **2.2 Proportion of ulcers completely healed(Sensitivity analysis)** | 7 | 452 | Risk Ratio (M-H, Random, 95% CI) | 1.69 [1.32, 2.15] |
| **2.3 Time to complete ulcer healing (day)** | 4 | 179 | Std. Mean Difference (IV, Random, 95% CI) | -0.84 [-1.15, -0.53] |
| **2.3.1 Short term follow-up** | 2 | 93 | Std. Mean Difference (IV, Random, 95% CI) | -0.80 [-1.22, -0.37] |
| **2.3.2 unkown follow-up period** | 2 | 86 | Std. Mean Difference (IV, Random, 95% CI) | -1.03 [-1.90, -0.16] |
| **2.4 Amputation** | 2 | 196 | Risk Ratio (M-H, Random, 95% CI) | 4.67 [0.02, 893.31] |
| **2.4.1 Long term follow-up** | 1 | 171 | Risk Ratio (M-H, Random, 95% CI) | 5.18 [0.25, 106.25] |
| **2.4.2 Unknown follow-up period** | 1 | 25 | Risk Ratio (M-H, Random, 95% CI) | 0.46 [0.05, 4.46] |
| **2.5 Total adverse events** | 2 | 234 | Risk Ratio (M-H, Random, 95% CI) | 0.73 [0.17, 3.15] |
| **2.5.1 Short term follow-up** | 1 | 63 | Risk Ratio (M-H, Random, 95% CI) | 0.29 [0.07, 1.31] |
| **2.5.2 Long term follow-up** | 1 | 171 | Risk Ratio (M-H, Random, 95% CI) | 1.33 [0.71, 2.50] |
| **2.6 Serious adverse events** | 3 | 265 | Risk Ratio (M-H, Random, 95% CI) | 0.82 [0.20, 3.34] |
| **2.6.1 Short term follow-up** | 1 | 69 | Risk Ratio (M-H, Random, 95% CI) | 0.57 [0.25, 1.32] |
| **2.6.2 Long term follow-up** | 1 | 171 | Risk Ratio (M-H, Random, 95% CI) | 3.63 [0.78, 16.95] |
| **2.6.3 Unknown follow-up period** | 1 | 25 | Risk Ratio (M-H, Random, 95% CI) | 0.23 [0.03, 1.79] |
| **2.7 Serious adverse events(Sensitivity analysis)** | 3 | 284 | Risk Ratio (M-H, Random, 95% CI) | 0.85 [0.22, 3.38] |

CI=95% confidence intervals
