# Supplementary material for: Heparin and Related Substances for Treating Diabetic Foot Ulcers: A Systematic Review and Meta-Analysis
Source: Front Endocrinol (Lausanne). 2022 Feb 24;13:749368. doi: 10.3389/fendo.2022.749368 (PMC8907383; doi:10.3389/fendo.2022.749368)
Supplement: Supplementary file 5 [file DataSheet_1.docx]

**Supplementary Inf 1.** Medline Searching strategy

1. exp Glycosaminoglycans/
2. mucopolysaccharide*.tw.
3. (chondroitin or dermatan* or heparin* or LMWH or hyaluronic acid or dalteparin or tedelparin or kabi?2165 or fragmin or FR?860 or nadroparin* or fraxiparin* or CY?216 or enoxaparin or PK?10169 or EMT?967 or lovenox or clexane or EMT?966 or ardeparin or normiflo or wy?90493 or bemiparin or certoparin or mono-embolex or alphaparin or sandoparin or danaproid or lomoparan or org?10172 or orgaran or fondaparinux or quixidar or arixtra or idraparinux or parnaparin or CB-01-05-MMX or fluxum or parvoparin or tinzaparin or innohep or Logiparin or reviparin* or LU?47311 or Clivarin* or galactosaminoglycan* or perlecan or polysialic acid or proteoheparan* or sulodexide* or syndecan or trichosaccaride*).tw.
4. (keratan adj (sulfate* or sulphate*)).tw.
5. or/1-4
6. exp Foot Ulcer/
7. exp Diabetic Foot/
8. (diabet* adj3 ulcer*).tw.
9. (diabet* adj3 (foot or feet)).tw.
10. (diabet* adj3 wound*).tw.
11. or/6-10
12. (5 and 11)

13. randomized controlled trial.pt.
14. controlled clinical trial.pt.
15. randomi?ed.ab.
16. placebo.ab.
17. clinical trials as topic.sh.
18. randomly.ab.
19. trial.ti.
20. or/13-19
21. exp animals/ not humans.sh.
22. 20 not 21
23. 12 and 22
