# Supplementary material for: Heparin and Related Substances for Treating Diabetic Foot Ulcers: A Systematic Review and Meta-Analysis
Source: Front Endocrinol (Lausanne). 2022 Feb 24;13:749368. doi: 10.3389/fendo.2022.749368 (PMC8907383; doi:10.3389/fendo.2022.749368)
Supplement: Supplementary file 6 [file DataSheet_2.docx]

**Supplementary Inf 2.** Rational of excluding studies

| **Study** | **Reason for exclusion** |
| --- | --- |
| Marin 2020 | Not people with diabetic foot ulcers |
| Braun 2014 | Review |
| Cesarone 2011 | Not people with diabetic foot ulcers |
| Ciccone 2014 | Review |
| Kalani 2004 | Editorial |
| Omole 2012 | Case report |
| Seidel 1991 | Controlled clinical trial, but not randomised |
| Shou 2008 | Did not measure any outcome of interest |
| Zhou 2004 | Did not measure any outcome of interest |

**Exclude studies**

Marin S, Popovic-Pejicic S, Radosevic-Caric B, Trtić N, Tatic Z,Selakovic S.Hyaluronic Acid Treatment Outcome on the Post-Extraction Wound Healing in Patients With Poorly Controlled Type 2 Diabetes: A Randomized Controlled Split-Mouth Study.Randomized Controlled Trial Med Oral Patol Oral Cir Bucal. 2020,25(2):e154-e160. doi: 10.4317/medoral.23061.

Braun LR, Fisk WA, Lev-Tov H, Kirsner RS, Isseroff RR. Diabetic foot ulcer: an evidence-based treatment update. American Journal of Clinical Dermatology. 2014, 15(3):267-87.

Cesarone MR, Incandela L, Belcaro G, De Sanctis MT, Ricci A, Griffin M. Two-week topical treatment with essaven gel in patients with diabetic microangiopathy: a placebo-controlled, randomized study. Angiology. 2001, 52(Suppl 3):S43-8.

Ciccone MM, Cortese F, Corbo F, Corrales NE, Al-Momen AK, Silva A, et al. Bemiparin, an effective and safe low molecular weight heparin: a review. Vascular Pharmacology.2014, 62(1):32-7.

Kalani M, Apelqvist J, Blomback M. Dalteparin improved chronic foot ulcers and reduced the number of amputations in diabetic peripheral arterial occlusive disease. Evidence-Based Medicine. 2004, 9(3):73.

Omole MK, Bello DE. Case studies assessment on rational use of drugs among patients with ischemic heart disease at a tertiary hospital in south west Nigeria. Asian Journal of Pharmaceutical and Clinical Research. 2012, 5(Suppl 1):20-5.

Seidel C, Richter UG, Bühler S, Hornstein OP. Drug therapy of diabetic neuropathic foot ulcers: transvenous retrograde perfusion versus systemic regimen. VASA. Zeitschrift fur Gefasskrankheiten. Journal for Vascular Diseases. 1991, 20(4):388-93.

Shou L, Ning L, Zhang QL. Observation of low molecular weight heparin in treatment of diabetic foot. Journal of Medical Research.2008,37(10):92-4.

Zhou L, He LB. Clinical observation of low molecular weight heparin in the treatment of diabetic foot. Chinese General Practice. 2004, 7(14):1096.
